# Learning curves of theta/beta neurofeedback in children with ADHD

**DOI:** 10.1007/s00787-016-0920-8

**Published:** 2016-11-19

**Authors:** Tieme W. P. Janssen, Marleen Bink, Wouter D. Weeda, Katleen Geladé, Rosa van Mourik, Athanasios Maras, Jaap Oosterlaan

**Affiliations:** 1 Clinical Neuropsychology Section, Vrije Universiteit, Van Der Boechorststraat 1, 1081 BT Amsterdam, The Netherlands; 2 Department of Psychology, Leiden University, Wassenaarseweg 52, 2333 AK Leiden, The Netherlands; 3 Yulius Academy, Dennenhout 1, 2994 GC Barendracht, The Netherlands; 4 Royal Dutch Kentalis, Vlampijpstraat 78, 3534 AR Utrecht, The Netherlands

**Keywords:** Neurofeedback, Theta/beta-training, ADHD, Paediatric, EEG, Learning curves

## Abstract

Neurofeedback is widely applied as non-pharmacological intervention aimed at reducing symptoms of ADHD, even though efficacy has not been unequivocally established. Neuronal changes during the neurofeedback intervention that resemble learning can provide crucial evidence for the feasibility and specificity of this intervention. A total of 38 children (aged between 7 and 13 years) with a DSM-IV-TR diagnosis of ADHD, completed on average 29 sessions of theta (4–8 Hz)/beta (13–20 Hz) neurofeedback training. Dependent variables included training-related measures as well as theta and beta power during baseline and training runs for each session. Learning effects were analyzed both within and between sessions. To further specify findings, individual learning curves were explored and correlated with behavioral changes in ADHD symptoms. Over the course of the training, there was a linear increase in participants’ mean training level, highest obtained training level and the number of earned credits (range *b* = 0.059, −0.750, *p* < 0.001). Theta remained unchanged over the course of the training, while beta activity increased linearly within training sessions (*b* = 0.004, 95% CI = [0.0013–0.0067], *p* = 0.005) and over the course of the intervention (*b* = 0.0052, 95% CI = [0.0039–0.0065], *p* < 0.001). In contrast to the group analyses, significant individual learning curves were found for both theta and beta over the course of the intervention in 39 and 53%, respectively. Individual learning curves were not significantly correlated with behavioral changes. This study shows that children with ADHD can gain control over EEG states during neurofeedback, although a lack of behavioral correlates may indicate insufficient transfer to daily functioning, or to confounding reinforcement of electromyographic activity. Clinical Trials Registration: This trial is registered at the US National Institutes of Health (ClinicalTrials.gov, ref. no: NCT01363544); https://clinicaltrials.gov/show/NCT01363544.

**Electronic supplementary material:**

The online version of this article (doi:10.1007/s00787-016-0920-8) contains supplementary material, which is available to authorized users.

## Introduction

Attention deficit hyperactivity disorder (ADHD) is a neuropsychiatric disorder that is characterized by a persistent pattern of inattentive and/or hyperactive and impulsive behavior that interferes with normal social, academic or occupational functioning [1, 2]. Currently, the most commonly applied intervention for ADHD is treatment with stimulant medication. Although stimulant medication is effective in short-term symptom reduction [3, 4], there is limited knowledge on long-term effectiveness and possible long-term negative effects [5]. Therefore, parents may be reluctant to agree with stimulant treatment for their child [6]. This situation urges the need for effective alternatives to pharmacological interventions. However, few alternative interventions prove efficacious when only probably blinded results are considered [7], which might indicate a considerable contribution of non-specific effects to the positive outcomes reported for alternative interventions. Research into working mechanisms of alternative interventions may help to distinguish effective from non-effective treatment elements, which may contribute to improving and developing non-pharmacological interventions for children with ADHD.

Neurofeedback is considered a promising non-pharmacological alternative for the treatment of ADHD. The most commonly used type of neurofeedback is based on electroencephalogram (EEG) activity of the patient and is, therefore, also called EEG biofeedback. During neurofeedback training, patients receive feedback of their current brain activity. Using operant conditioning principles, the aim is that patients learn to modify the activation state of their brain. Children with ADHD may have atypical EEG power spectra, showing increased theta and decreased beta activity [8, 9]. On a behavioral level, theta has been negatively related to alertness, whereas beta has been positively related to attention [10–12]. Accordingly, one of the most commonly applied neurofeedback protocol aims to decrease theta (4–8 Hz) activity and increase beta (13–20 Hz) activity [12–15]. Behavioral outcomes for theta/beta neurofeedback in children with ADHD vary across studies. Whereas unblinded randomized controlled trials (RCT) reveal superiority of neurofeedback over control conditions [16, 17], blinded placebo-controlled studies show similar improvements in behavior for neurofeedback and sham-neurofeedback [18–20].

Surprisingly, data on training progress within and between neurofeedback sessions are scarcely considered in ADHD [21] and the available results are mixed. Uncontrolled studies showed that a majority of children with ADHD who displayed elevated theta/beta ratios before the training were able to decrease theta [22] or theta/beta ratio over the course of theta/beta neurofeedback training [23]. In contrast, another study showed that children with ADHD were not able to decrease theta/beta ratios over the training sessions [24]. Only two RCT studies reported on training data of theta/beta protocols. The first study of Vollebregt, van Dongen-Boomsma, Buitelaar, and Slaats-Willemse [20] analyzed a small subsample of ten children who received different forms of theta/beta neurofeedback. Seven out of ten children showed the desired change over time in either theta or beta power. However, all ten children showed also changes in one frequency band in the opposite of the trained direction. The second study [25] showed that adolescents who received theta/sensorimotor rhythm neurofeedback (SMR; 12–15 Hz) became better in suppressing theta within training sessions. However, the mean value of theta did not decrease over the entire treatment. Both studies [20, 25] reported behavioral improvements over time, irrespective of whether the children received an active form of theta/beta neurofeedback training or not. In conclusion, the current literature remains ambiguous to what extend theta and beta activity can be trained in children with ADHD and whether such training effects underlie the behavioral changes observed.

Other important aspects of neurofeedback concern stability and generalizability of trained frequencies. If children with ADHD are able to adapt theta or beta activity, the question is whether this leads to sustainable changes that also generalize to situations outside neurofeedback training sessions. Two RCT studies indeed reported a linear decrease in theta activity at midline scalp electrodes after the training was completed [26, 27]. In contrast, another study did not find any differences in theta or beta activity for children following individualized theta/beta neurofeedback [28]. Alternatively, when it seems that neurofeedback results mainly in state changes, it is of clinical importance to know whether this temporary state can be generalized to daily activities such as school activities. To promote generalizability to daily life, some neurofeedback training programs apply transfer trials. During these trials, patients receive feedback on their performance after the trial is completed and do not receive feedback during the trial. Additionally, transfer cards, with a visual representation of the neurofeedback training screen, may be given to patients to use in daily life. Transfer cards are supposed to evoke the desired decreased theta/beta ratio as a conditioned response and may thus be used to improve attention, for example during school assignments.

In summary, several studies investigated the behavioral and neurocognitive outcomes of theta/beta neurofeedback; however, little is known about specific working mechanisms of theta/beta neurofeedback. Therefore, the aim of the current study was to explore the effects of theta/beta neurofeedback on EEG activity within and between neurofeedback sessions over the course of a 10-week, 30 sessions theta/beta neurofeedback intervention in children with ADHD. More specifically, both training parameters between sessions (mean and maximum training level), and theta and beta power within and between training sessions, were tested for learning effects. Beside these group analyses, we identified individual learners as participants that were able to significantly change theta or beta power in the desired direction. Generalizability was explored by comparing transfer and non-transfer trials, and baseline versus training EEG. Additionally, we explored whether the ability to adapt EEG theta or beta activity was related to ADHD symptom reductions.

## Materials and methods

### Participants

This study pertains to a subsample of 38 children that were randomized to neurofeedback intervention in a multicenter three-way parallel group study with balanced randomization into the effects of neurofeedback compared to optimally titrated methylphenidate and physical activity (applied as a semi-active control condition) in children with ADHD (Clinical Trials Registration: ClinicalTrials.gov, Identifier: NCT01363544). Of the 38 children, 7 had comorbid disorders including learning disorders (*n* = 3), autism spectrum disorders (ASS; *n* = 2), learning disorder combined with ASS (*n* = 1), and learning disorder combined with anxiety disorder (*n* = 1). Group characteristics are listed in Table 1. For further details, please see Janssen et al. [27].

**Table 1:** Group characteristics

	*N*	Mean	SD	Range
Age	0038	9.87	1.81	7.2 to 13.6
Gender (male/female)	0038	29/9		
IQ	0038	100.45	13.34	81 to 134
DBDRS parent				
Inattention	0038	16.63	5.15	8 to 26
Hyperactivity/impulsivity	0038	14.50	5.99	3 to 25
DBDRS teacher				
Inattention	0038	15.37	5.29	4 to 25
Hyperactivity/impulsivity	0038	13.79	6.90	3 to 26
SWAN parent				
Inattention	0038	1.44	0.51	0.22 to 2.33
Hyperactivity/impulsivity	0038	1.30	0.71	−0.89 to 2.33
SWAN teacher				
Inattention	0037	1.37	0.91	−0.67 to 2.89
Hyperactivity/impulsivity	0037	1.15	0.92	−1.11 to 2.78

*DBDRS* disruptive behavior disorders rating scale (raw scores), strengths and weaknesses of ADHD symptoms and normal behavior scale (SWAN), *SD* standard deviation

### Neurofeedback intervention

**Fig. 1:**
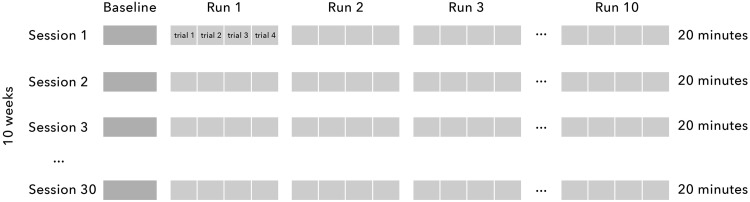
Schematic representation of the neurofeedback intervention. Approximately 3 sessions of neurofeedback were provided in a week, with 30 sessions taking 10 weeks. Each neurofeedback session started with a 1-min theta/beta baseline recording, which was used for the remaining of the session. A session consisted of 10 runs of 2 min each (20 min effective training). Each run comprised 4 trials of 30 s. Children were instructed to decrease theta/beta compared to the baseline recording during each trial. Children were rewarded with credits related to the size of the improvements compared to baseline. The training level increased or decreased based on performance of former runs and ranged between 3 and 52%. Training level increased after two successive runs with three successful trials each or after one run with four successful trials. Training level decreased after two successive runs with only one successful trial or after one run with no successful trials

Neurofeedback consisted of three individual training sessions a week, with each session lasting 45 min including 20 min of effective training, over a period of 10 to 12 weeks. The mean number of completed training sessions was 29 (*M* = 28.53, SD = 2.63, range 19–30). Each training session consisted of 10 runs and started with a 1-min baseline theta/beta index measurement. This baseline measurement was used in the following 10 runs of neurofeedback. In sum, theta and beta were recorded during these runs and children were rewarded with credits related to the size of the improvements compared to baseline. See Fig. 1 for a schematic representation of the neurofeedback intervention.

In more detail, theta/beta training was applied unidirectionally, with the aim to inhibit theta (4–8 Hz) and reinforce beta (13–20 Hz) activity at Cz. The THERAPRAX^**®**^ EEG Biofeedback system (Neuroconn GmbH, Germany) with a DC-amplifier and a sampling rate of 128 Hz was used to transmit and analyze the EEG signal. Reference and ground electrodes were attached to right and left mastoids, respectively. Electro-oculogram (EOG) was obtained with two electrodes at external canthi, and two electrodes at infra- and supra-orbital sides. Ocular correction was applied as described in Schlegelmilch et al. [29]. Subsequently, theta/beta index [theta(μV^2^/Hz) − beta(μV^2^/Hz)/theta(μV^2^/Hz) + beta(μV^2^/Hz)] was computed with a short-time-fourier transformed moving average for direct feedback during runs.

Each run comprised four 30-s trials. The first run of the first session started on a training level with the aim to reduce the theta/beta index with 3% compared to baseline. The training level increased or decreased based on performance of former runs and ranged between 3 and 52%. Training level increased after two successive runs with three successful trials each or after one run with four successful trials. Training level decreased after two successive runs with only one successful trial or after one run with no successful trials. From the second session onwards, training level of the first run was set at the second lowest level that was achieved in the former session. Theta/beta index was represented to the participant by simple graphics on a screen. Successful reduction of the theta/beta index as averaged over one trial relative to the baseline was rewarded with the appearance of a sun and granted with credits. The number of earned credits per trial depended on the training level, with more credits for higher levels to reinforce and motivate children to increase performance: 3 and 5% were rewarded with 1 credit; 10, 12, and 14% were rewarded with 2 credits; 16, 19, and 23% were rewarded with 3 credits; 26, 28, and 30% were rewarded with 4 credits; 33, 35, and 37% were rewarded with 5 credits; 40, 42, 45, and 47% were rewarded with 6 credits, and 50 and 52% were rewarded with 7 credits.

Transfer trials without immediate visual feedback were included from session 11 (25%) and session 21 (50%) onwards. To further transfer learned behaviors, participants were instructed to retrieve their neurofeedback experiences by watching printed graphics of the training during school and homework. Compliance was verified by questioning the participants whether they used the transfer cards over the intervention period. Transfer cards were used by 84% of the participants.

### Outcome measures

#### Training outcomes

During the neurofeedback sessions the trainer kept track of the training level represented as the percentage decrease in theta/beta index as compared to baseline for each run separately as well as the total number of earned credits per run. Dependent measures included the mean training level over runs within a session (%), the best run of each session (maximum achieved training level), and the total number of obtained credits per session.

#### EEG

EEG-recordings were analyzed with Brain Vision Analyzer v2.0 (Brain Products GmbH, Germany). A high-pass filter of 0.5 Hz, 12 dB/octave and a low-pass filter of 30 Hz, 48 dB/octave were applied. Ocular correction was applied as in Gratton, Coles, and Donchin [30]. Data of the training baselines and runs were segmented in 2-s epochs. Automatic raw data inspection was applied with a maximum allowed voltage step between samples of 50 μV/ms, maximum allowed difference of 120 μV in each segment, and permitted amplitude range of −100, 100 μV. Before and after detected artifacts, 200 ms of data were removed. The lowest permitted activity in intervals was 0.5 μV with an interval length of 50 ms. Fast Fourier Transformation (FFT) with a 20% Hamming window was applied for tapering, and averages over the artifact-free epochs were calculated. Mean power (μV^2^) was exported to SPSS for the trained frequency bands: theta 4–8 Hz, and beta 13–20 Hz. EEG baseline was available for 93 and 92% of the training data, respectively.

#### Behavior

Raw scores on the scales Inattention and Hyperactivity/Impulsivity of the Strengths and Weaknesses of ADHD symptoms and Normal behavior scale (SWAN) [31] were used to evaluate the relation between behavioral change and theta- and beta-slopes within and between sessions (see Statistical Analysis).

### Procedure

Prior to the start of the study, approval was obtained from the national medical ethics committee (Ref. no: NL 31641.029.10 CCMO). Children were recruited from 15 child mental health institutions in the West of the Netherlands. Written informed consent was obtained from the parents and children aged 11 years and older. Interventions took place between September 2010 and March 2014. The duration of the intervention period was approximately 10 weeks.

### Statistical analysis

All analyses were performed using SPSS version 21.0 (Corp IBM, released 2012). Values of *p* < 0.05 were considered statistically significant. Only significant results are reported. For all outcome measures data were available for at least 92% of the participants.

Linear mixed models were used to test whether children with ADHD were able to learn to adapt EEG theta and beta activity within and between neurofeedback sessions over the course of a 10-week, 30 sessions theta/beta neurofeedback intervention. Dependent variables included training outcomes as well as theta and beta power. The linear model included linear fixed effects for session and run, a random intercept over participants, and a random slope for session and run. Exploratory analyses were conducted to assess possible differences in the distribution of transfer trials and feedback trials over the sessions, which were performed with the addition of the fixed factor percentage of transfer trials and the interaction of percentage of transfer trials with session. Additionally, to test whether a parabolic function would increase the fit of the model in addition to linear functions, analyses were performed with the addition of quadratic terms for run and session. Finally, because of possible effects of comorbid disorders on the theta/beta ratio [32, 33], sensitivity analyses were performed with exclusion of the children with ADHD and comorbid disorders (*n* = 7).

To explore individual learning curves in the ability to adapt EEG theta or beta activity within and between 30 neurofeedback sessions, separate mixed models were performed for theta and beta power for each participant. The linear model included linear fixed effects for session and run. For each participant, the individual slopes over runs and sessions were used to determine whether there was a significant decrease in theta power and an increase in beta power. Learners were defined as participants able to significantly change theta or beta power in the desired direction.

To explore whether the individual ability to adapt EEG theta or beta activity was related to behavioral changes, linear stepwise regression models were performed with SWAN difference scores (SWAN scores post-intervention minus pre-intervention) as dependent variables. Two sets of independent variables were analyzed: (1) theta slopes over runs and theta slopes over sessions and (2) beta slopes over runs and beta slopes over sessions. SWAN teacher scores were missing for one participant.

## Results

### Training outcomes

Outcome measures for the total group are listed in Table 2. Mean training level increased linearly over the training sessions, *F*(1,1040.164) = 34.61, *p* < 0.001, *b* = 0.0877, 95% CI = [0.0585 to 0.1171], and was accompanied by a linear increase in the maximum obtained training level over the training sessions, *F*(1,1041.022) = 29.04, *p* < 0.001, *b* = 0.1150, 95% CI = [0.0731 to 0.1568]. Total number of gained credits also increased over sessions, *F*(1,1042.525) = 58.08, *p* < 0.001, *b* = 0.7501, 95% CI = [0.5570 to 0.9433].

**Table 2:** Outcome measures

	Available data		Mean	SD	Range
	*N*	%			
Number of sessions followed	38		28.53	2.63	19.00 to 30.00
Training outcomes					
Mean training level per session (%)	1076	99	6.64	5.61	3.00 to 46.20
Max training level per session (%)	1076	99	9.94	7.42	3.00 to 52.00
Amount of gained credits per session	1076	99	28.22	32.18	0.00 to 220.00
EEG theta activity					
Mean theta baseline (μV^2^)	1007	93	1.71	0.40	0.30 to 3.78
Mean theta training (μV^2^)	10002	92	1.67	0.46	0.30 to 4.72
EEG beta activity					
Mean beta baseline (μV^2^)	1007	93	0.67	0.16	0.21 to 1.78
Mean beta training (μV^2^)	10002	92	0.75	0.22	0.19 to 2.50
SWAN parent difference scores: pre- to post-intervention					
Inattention	38	100	−0.32	0.66	−2.22 to 0.78
Hyperactivity/impulsivity	38	100	−0.29	0.68	−1.67 to 1.44
SWAN teacher difference scores: pre- to post-intervention					
Inattention	37	97	−0.11	0.66	−1.44 to 1.89
Hyperactivity/impulsivity	37	97	−0.03	0.82	−2.33 to 1.78

Strengths and Weaknesses of ADHD symptoms and Normal behaviour scale (SWAN)

* SD* standard deviation

### EEG learning curves

#### Theta activity

Training baseline theta activity did not change over the course of the sessions, *F*(1, 969.765) = 0.412, *p* = 0.521, *b* = −0.0006, 95% CI = [−0.0023 to 0.0012]. During the intervention, theta activity did not change over sessions, *F*(1, 44.687) = 0.322, *p* = 0.573, *b* = −0.0020, 95% CI = [−0.0090 to 0.0050]. Furthermore, theta activity did not change over runs within sessions, *F*(1, 1040.445) = 1.844, *p* = 0.175, *b* = −0.0010, 95% CI = [−0.0004 to 0.0023].

#### Beta activity

**Fig. 2:**
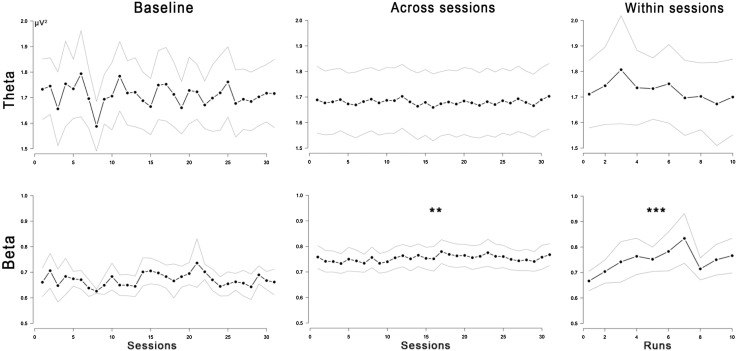
EEG learning effects during neurofeedback. These graphs show changes in theta and beta power (μV^2^) during 1-min baseline EEG recordings preceding each neurofeedback session, across sessions (1–30) and within sessions (runs 1–10). The *bold lines* are means, and the *upper* and *lower lines* are 95% confidence intervals. **p < 0.01, ***p < 0.001

Similar to theta, beta activity during training baseline did not change over the sessions, *F*(1, 971.583) = 1.87, *p* = 0.171, *b* = 0.0007, 95% CI = [−0.0003 to 0.0016]. In contrast, during the intervention, beta activity showed a linear increase over sessions, *F*(1, 57.461) = 8.60, *p* = 0.005, *b* = 0.0040 95% CI = [0.0013 to 0.0067]. Additionally, beta activity increased linearly over runs within sessions, *F*(1, 1012.625) = 63.51, *p* < 0.001, *b* = 0.0052 95% CI = [0.0039 to 0.0065]. EEG learning curves are shown in Fig. 2.

### Exploratory analyses

Analyses showed that the percentage of transfer trials during a session did not significantly influence training results in theta and beta bands. Furthermore, quadratic terms did not further improve the linear mixed models. Sensitivity analyses showed that results remained essentially unchanged when analyses were rerun, excluding children with ADHD and comorbid disorders (*n* = 7), indicating that the presence of comorbid disorders did not affect the results. Only a near-significant effect for baseline beta was found, showing a slight increase over the sessions with exclusion of comorbid disorders, *F*(1, 796.367) = 3.862, *p* = 0.050, *b* = 0.00095, 95% CI = [0.000001 to 0.001903]. Complete statistical details are provided in Supplementary Table 1.

### Individual learning curves

#### Theta activity

Over the course of the intervention, 23 (61%) participants showed a negative slope for theta over the sessions of which 15 (39%) participants showed a significant negative slope and were indicated as theta session learners. However, 7 (18%) participants showed a significant change over the sessions in the opposite of the desired direction with an increase in theta. Over the runs within sessions, 14 (37%) of the participants presented a negative slope, although only 4 (11%) of these participants showed a significant negative slope and were indicated as theta run learners. Only one (3%) participant showed a significant positive theta slope over runs.

#### Beta activity

A total of 28 (74%) participants showed the desired positive beta slope over sessions, of which 20 (53%) participants showed a significant positive slope and were indicated as beta session learners. Over the sessions, 3 (8%) participants showed a significant negative beta slope. Thirty-one (82%) participants were able to increase beta over runs within sessions and 13 (34%) participants were indicated as beta run learners (showing a significant positive slope). There were no participants that showed a significant negative beta slope over runs.

### Behavioral change and EEG slopes

No significant associations were found between beta or theta individual slopes over sessions or runs and changes in SWAN Inattention and Hyperactivity/impulsivity scales (post- minus pre-intervention) as reported by parents and teachers. Complete statistical details are provided in Supplementary Table 2.

## Discussion

Theta/beta neurofeedback is aimed at altering brain activity using operant conditioning principles with the goal to improve behavior and neurocognitive functioning in children with ADHD. However, few studies have demonstrated that actual learning takes place during neurofeedback treatment in children with ADHD, which is an essential element for the effectivity of the intervention. The results of the current study provide evidence that children with ADHD learned to decrease the theta/beta index over the sessions in a linear fashion, concordant with the training goals. More detailed analysis of electroencephalogram (EEG) data obtained during the intervention seems to ascribe learning effects primarily to increasing beta power over and within sessions at group level, and at the individual level as well. However, for the theta band, analyses of individual EEG learning curves showed considerable interindividual variation, masking potential effects at group level.

An important general aspect of neurofeedback is a correct translation of learning theory principles to the training design [34]. The current study complies with most of the suggested relevant learning principles, such as proper timing of feedback (<250 ms), specificity of the trained EEG signal (online EOG correction), shaping (adjusting thresholds and reinforcement magnitude), type of reinforcement (simple graphic), and generalization (successful application of transfer trials and transfer cards), which are probably reflected in the successful training results. It should be noted that this and other comparable studies still have a way to go concerning the specificity of the trained signal. First, knowledge about the functional meaning of theta/beta ratio should be improved. Second, artifacts, such as electromyographic (EMG) activity produced by skeletal muscles, may covertly influence theta/beta ratio [35, 36].

In the theta band, we could not demonstrate learning effects at the group level; however, more detailed analysis of individual learning curves showed considerable heterogeneity in training results. While 15 participants significantly learned to reduce theta in accordance with the training goals (39%), 7 children (18%) increased theta and 16 children (43%) showed no statistically significant training effects over sessions. Only the study by Lubar et al. [22] found comparable results as in our study, showing 12 significant learners (63%) over sessions. We identified only few learners in the within-session data, with 4 significant learners in line with the training goals (11%), and 1 child showing training effects in the opposite direction. One other study by Bink et al. [25] demonstrated successful suppression of theta within training sessions. An alternative explanation for theta decreases in our study may be the fact that children show a developmental decrease in theta activity over time [37, 38], with a drop in theta activity around the age of nine years [38]. Since the majority was not able to suppress theta within sessions, it is questionable whether the negative individual theta slopes over the intervention indeed result from the neurofeedback intervention or rather originate from developmental changes.

Learning effects were most apparent in the beta frequency band, with linear beta power increases over and within 30 sessions of neurofeedback at group level. At the individual level, more than half of the children were identified as significant beta learners over the sessions, and approximately one-third as significant beta learners over the runs within the sessions. It is difficult to establish whether beta changes represent genuine alterations in brain activity or reflect the reinforcement of artifact data, such as EMG activity [35, 36]. Although the peak frequency of EMG is at relatively high frequencies, the EMG spectrum is very broad and may influence adjacent beta frequencies more than lower frequency bands such as theta. Despite specific instructions during neurofeedback training to prevent excessive muscular tension, it cannot be ruled out that some children used more subtle covert muscular tension to influence the theta/beta ratio. This explanation is further supported by the fact that we could not convincingly demonstrate increases in baseline beta, as recorded before commencing each neurofeedback training, and the lack of chronic beta changes during both rest and task conditions at post-measurement [39]. To reduce potential EMG contamination, higher frequencies may be inhibited, such as in the Lubar protocol [40], or EMG activity could be separately monitored for the major skeletal muscles.

A challenging task is to interpret the various electrophysiological findings of this study. Although we demonstrated learning effects in theta and beta frequency bands, these were not significantly related to symptom improvement in children with ADHD, which may be a necessary prerequisite to demonstrate the specificity of the results [21]. Furthermore, the behavioral findings [41] could not confirm the efficacy of theta/beta neurofeedback compared to the control group according to parent and teacher reports. Surprisingly, at post-treatment, children that received theta/beta neurofeedback were characterized by a specific decrease in theta power compared to the control group during a resting eyes open condition, but not during a task condition [27]. This pattern of results may represent chronic effects of the intervention, which do not generalize to a task- and goal-related context, consistent with a lack of event-related potential (ERP) effects during the same task [42]. It may, therefore, be contradictory that in the current study no effects were found for the baseline power spectra measurements, which were performed at the start of each neurofeedback training. This might be explained by the fact that these measurements were of shorter duration, mostly in the afternoon instead of the morning, and in a different context.

A few limitations should be considered when interpreting the findings of the current study. First, although the study contained a random sample of children with ADHD due to the RCT design, a relative large participant sample (*n* = 38), and large number of EEG data points (30 sessions × 10 trials; approximately 10 h for each participant), the design of the study did not allow to compare neurofeedback with a placebo-controlled condition. Accordingly, this precludes stronger conclusions on the specificity of the findings. Future sham-controlled studies could assess whether theta and beta changes between and within sessions are due to developmental changes and EMG artefacts or not. Second, although training and EEG analyses solely involved the vertex location on the scalp, neurofeedback may alter a more extended cortical region. Future studies may add more electrodes to measure widespread EEG effects during the intervention, while still confining the feedback signal to the vertex. Third, a noticeable qualitative observation of within-session beta effects is a steep decline in beta power at approximately 3/4 of the session (run 8 out of 10) after a linear increase in beta power (run 1 to 7; see also Fig. 1). This might indicate that children were not able to remain motivated towards the end of the session or that they could not sustain the energy demands of the training. Future studies may consider these factors, especially considering potential detrimental effects on acquired learning in the first part of the training. Last, cost-effectiveness of neurofeedback could be increased with predictive models of treatment success. Although learning curves were not predictive in the current study, theta power at pre-treatment has been found predictive [27]. It would be worthwhile to search for additive predictive factors.

Overall, the results of the current study show that learning took place during theta/beta neurofeedback in children with ADHD. However, it remains more difficult to interpret these findings, especially since training results were not related to behavioral changes. Future studies are encouraged to obtain electrophysiological training data and to report on the various training components of the intervention. This kind of data can play an important role in developing more effective neurofeedback interventions for ADHD, by isolating trainable components and improving our understanding of the underlying mechanisms of neurofeedback.

## Electronic supplementary material

Below is the link to the electronic supplementary material.

Supplementary material 1 (DOCX 20 kb)
