# Supplementary material for: Learning curves of theta/beta neurofeedback in children with ADHD
Source: Eur Child Adolesc Psychiatry. 2016 Nov 19;26(5):573–82. doi: 10.1007/s00787-016-0920-8 (PMC5394134; doi:10.1007/s00787-016-0920-8)
Supplement: Supplementary file 1 — Supplementary material 1 (DOCX 20 kb) [file 787_2016_920_MOESM1_ESM.docx]

**Supplementary material**

| **Supplementary Table 1.** Complete results mixed effects models and sensitivity analyses | | | | | | | | |
| --- | --- | --- | --- | --- | --- | --- | --- | --- |
|  | **Session** | | | | **Run** | | | |
|  | *df* |  | *F* | *p* | *df* |  | *F* | *p* |
| Main analyses (*n*=38) | | | | | | | | |
| Mean training level | 1,000 | 1040,164 | 34,610 | 0,000 | n/a | n/a | n/a | n/a |
| Max training level | 1,000 | 1041,022 | 29,040 | 0,000 | n/a | n/a | n/a | n/a |
| Total number credits | 1,000 | 1042,525 | 58,076 | 0,000 | n/a | n/a | n/a | n/a |
| Baseline theta | 1.000 | 969.765 | 0.412 | 0.521 | n/a | n/a | n/a | n/a |
| Baseline beta | 1.000 | 971.583 | 1.874 | 0.171 | n/a | n/a | n/a | n/a |
| Training theta | 1.000 | 44.687 | 0.332 | 0.573 | 1.000 | 1040.445 | 1.844 | 0.175 |
| Training beta | 1.000 | 57.461 | 8.595 | 0.005 | 1.000 | 1012.625 | 63.514 | 0.000 |
|  | | | | | | | | |
| **Transfer trials** | **Percentage transfer trials** | | | | **Percentage transfer trials x Session** | | | |
| Training theta | 2.000 | 969.325 | 0.256 | 0.774 | 2.000 | 970.324 | 0.079 | 0.924 |
| Training beta | 2.000 | 850.507 | 0.753 | 0.471 | 2.000 | 851.700 | 1.032 | 0.357 |
| Sensitivity analyses (excluding comorbid disorders, *n*=31) | | | | | | | | |
|  | **Session** | | | | **Run** | | | |
| Mean training level | 1.000 | 859.688 | 10.178 | 0.001 | n/a | n/a | n/a | n/a |
| Max training level | 1.000 | 859.959 | 9.357 | 0.002 | n/a | n/a | n/a | n/a |
| Total number credits | 1.000 | 861.055 | 37.463 | 0.000 | n/a | n/a | n/a | n/a |
| Baseline theta | 1.000 | 794.428 | 0.351 | 0.554 | n/a | n/a | n/a | n/a |
| Baseline beta | 1.000 | 796.367 | 3.862 | 0.050 | n/a | n/a | n/a | n/a |
| Training theta | 1.000 | 36.554 | 0.348 | 0.559 | 1.000 | 854.321 | 2.329 | 0.127 |
| Training beta | 1.000 | 58.068 | 6.917 | 0.011 | 1.000 | 824.721 | 54.281 | 0.000 |
|  | | | | | | | | |
| **Transfer trials** | **Percentage transfer trials** | | | | **Percentage transfer trials x Session** | | | |
| Training theta | 2.000 | 793.450 | 0.246 | 0.782 | 2.000 | 794.278 | 0.212 | 0.809 |
| Training beta | 2.000 | 684.492 | 0.584 | 0.558 | 2.000 | 685.394 | 0.397 | 0.672 |

*Note. df* = degrees of freedom, n/a = not applicable.

| **Supplementary Table 2.** Correlations between individual learning curves (*b*) and ADHD symptom change (t1-t0) | | | | | |
| --- | --- | --- | --- | --- | --- |
|  |  | Parents | | Teacher | |
|  |  | IN (t1-t0) | H/I (t1-t0) | IN (t1-t0) | H/I (t1-t0) |
| Theta runs (*b*) | *r* | .022 | -.138 | .192 | .267 |
|  | *p* | .897 | .410 | .254 | .110 |
|  | *n* | 38 | 38 | 37 | 37 |
| Theta session (*b*) | *r* | -.196 | -.051 | -.076 | .016 |
|  | *p* | .238 | .761 | .653 | .925 |
|  | *n* | 38 | 38 | 37 | 37 |
| Beta runs (*b*) | *r* | -.274 | -.221 | .085 | .137 |
|  | *p* | .096 | .183 | .617 | .418 |
|  | *n* | 38 | 38 | 37 | 37 |
| Beta session (*b*) | *r* | -.034 | .112 | -.159 | -.014 |
|  | *p* | .840 | .503 | .346 | .936 |
|  | *n* | 38 | 38 | 37 | 37 |

*Note.* IN = inattention (SWAN questionnaire), HI = hyperactivity/impulsivity (SWAN), t0 = pre-intervention, t1 =

post-intervention, *b* = beta (slope, measure of change), *r* = Pearson correlation, *p* = p-value, *n* = number of observations
